# Agents contributing to secondary immunodeficiency development in patients with multiple myeloma, chronic lymphocytic leukemia and non-Hodgkin lymphoma: A systematic literature review

**DOI:** 10.3389/fonc.2023.1098326

**Published:** 2023-02-07

**Authors:** Stephen Jolles, Sergio Giralt, Tessa Kerre, Hillard M. Lazarus, S. Shahzad Mustafa, Roberto Ria, Donald C. Vinh

**Affiliations:** ^1^ Immunodeficiency Centre for Wales, University Hospital of Wales, Cardiff, United Kingdom; ^2^ Division of Hematologic Malignancies, Memorial Sloan Kettering Cancer Center, New York, NY, United States; ^3^ Faculty of Medicine and Health Sciences, Ghent University Hospital, Ghent, Belgium; ^4^ Department of Medicine, Hematology-Oncology, Case Western Reserve University, Cleveland, OH, United States; ^5^ Rochester Regional Health, Rochester, NY, United States; ^6^ Department of Medicine, Allergy/Immunology and Rheumatology, University of Rochester, Rochester, NY, United States; ^7^ Department of Biomedical Sciences and Human Oncology, University of Bari Aldo Moro Medical School, Bari, Italy; ^8^ Department of Medicine, McGill University Health Centre, Montreal, QC, Canada

**Keywords:** secondary immunodeficiency, hematological malignances, neutropenia, hypogammaglobulinemia, secondary antibody deficiency, B-lineage monoclonal antibodies, Bruton kinase inhibitors

## Abstract

**Introduction:**

Patients with hematological malignancies (HMs), like chronic lymphocytic leukemia (CLL), multiple myeloma (MM), and non-Hodgkin lymphoma (NHL), have a high risk of secondary immunodeficiency (SID), SID-related infections, and mortality. Here, we report the results of a systematic literature review on the potential association of various cancer regimens with infection rates, neutropenia, lymphocytopenia, or hypogammaglobulinemia, indicative of SID.

**Methods:**

A systematic literature search was performed in 03/2022 using PubMed to search for clinical trials that mentioned in the title and/or abstract selected cancer (CLL, MM, or NHL) treatments covering 12 classes of drugs, including B-lineage monoclonal antibodies, CAR T therapies, proteasome inhibitors, kinase inhibitors, immunomodulators, antimetabolites, anti-tumor antibiotics, alkylating agents, Bcl-2 antagonists, histone deacetylase inhibitors, vinca alkaloids, and selective inhibitors of nuclear export. To be included, a publication had to report at least one of the following: percentages of patients with any grade and/or grade ≥3 infections, any grade and/or grade ≥3 neutropenia, or hypogammaglobulinemia. From the relevant publications, the percentages of patients with lymphocytopenia and specific types of infection (fungal, viral, bacterial, respiratory [upper or lower respiratory tract], bronchitis, pneumonia, urinary tract infection, skin, gastrointestinal, and sepsis) were collected.

**Results:**

Of 89 relevant studies, 17, 38, and 34 included patients with CLL, MM, and NHL, respectively. In CLL, MM, and NHL, any grade infections were seen in 51.3%, 35.9% and 31.1% of patients, and any grade neutropenia in 36.3%, 36.4%, and 35.4% of patients, respectively. The highest proportion of patients with grade ≥3 infections across classes of drugs were: 41.0% in patients with MM treated with a B-lineage monoclonal antibody combination; and 29.9% and 38.0% of patients with CLL and NHL treated with a kinase inhibitor combination, respectively. In the limited studies, the mean percentage of patients with lymphocytopenia was 1.9%, 11.9%, and 38.6% in CLL, MM, and NHL, respectively. Two studies reported the proportion of patients with hypogammaglobulinemia: 0–15.3% in CLL and 5.9% in NHL (no studies reported hypogammaglobulinemia in MM).

**Conclusion:**

This review highlights cancer treatments contributing to infections and neutropenia, potentially related to SID, and shows underreporting of hypogammaglobulinemia and lymphocytopenia before and during HM therapies.

## 1 Introduction

### 1.1 Secondary immunodeficiency (SID) in patients with hematological malignancies (HMs)

SID is a group of disorders in which cell-mediated immunity and/or humoral immune responses are compromised by non-inherited factors, increasing the risk of infections (1, 2). SID can be caused by several factors, including non-genetic metabolic diseases (e.g., protein-losing enteropathy, diabetes mellitus, chronic kidney disease, etc.), malnutrition, medications and malignancies, among others (2–4). Patients with HMs, including chronic lymphocytic leukemia (CLL), multiple myeloma (MM), and non-Hodgkin lymphoma (NHL), have a higher risk of SID, SID-related infections, and mortality compared with immunocompetent individuals (3, 5). Their risk of developing SID and SID-related infections is influenced by the distinct intrinsic pathophysiology of the disease, the use of and exposure time to different cancer treatments, and the presence of certain comorbidities, such as chronic lung or heart disease, kidney failure, diabetes, chronic obstructive pulmonary disease, and hypertension, of which some are caused or aggravated by cancer treatments (1, 3, 5–8). Interestingly, differences exist in both the sites and pathogen spectrum associated with certain HMs and their treatments (9–13), which might also be different from those observed in primary immunodeficiency (PID) (14). Additionally, there is a growing body of evidence that suggests that PID-related genes might influence the development of certain HMs and the likelihood of SID development in cohorts of patients with HMs (15–19).

### 1.2 Agents contributing to SID development in patients with HMs

Various agents used to treat HMs have been reported to increase the risk of infection due to their mode of action or as associated adverse effects on the immune system that are not clearly related to the pharmacologic activity of the molecule (2, 5, 8). These agents can affect the innate and/or adaptive immune systems in different ways, depending on which component they target (e.g., neutrophils, dendritic cells [DC], granulocytes, monocytes, and macrophages, which regulate the innate immune response; antibodies, B and T cells, which regulate the adaptive immune response; natural killer cells [NK], which are involved in both the innate and adaptive immune responses) (20, 21).

Anti-cancer monoclonal antibodies can be detrimental to both the innate and the adaptive immune systems based on the antigens they target. For instance, anti-CD20 antibodies primarily induce B-cell depletion, since CD20 is expressed by B cells only. However, since CD52 is expressed by T cells, B cells, granulocytes, monocytes, macrophages, NK cells, and DC, monoclonal antibodies directed against CD52 will impact both the innate and adaptive immune systems (22, 23). In addition, monoclonal antibodies can lead to infections, neutropenia, and sometimes cause a prolonged delay of functional recovery of the targeted cell population (24–28). In a similar way, the effects of chimeric antigen receptor T-cell (CAR T) therapies on the immune systems are influenced by which antigens the T cells are engineered to target; but can also lead to other adverse events related directly to its mode of action (e.g., cytokine release syndrome and hypogammaglobulinemia) and other adverse effects considered ‘on-target off-tumor’, like infections, neutropenia, and fatigue (22, 24, 29).

Proteasome inhibitors can induce neutropenia, reduce the number of T cells, NK cells and DC, alter NK-cell and CD8+ T-cell function, and cytokine production, therefore affecting both innate and adaptive processes (1, 30). Several kinases are involved in both the proliferation, activation, and survival of malignant cells, as well as the regulation of signaling pathways of immune cells (e.g., granulocytes, monocytes, DC, and NK cells for the regulation of the innate immune response; antibody production, T and B cells for the regulation of the adaptive immune response) (31).

Kinase inhibitors have drastically helped manage HMs; however, they can compromise the correct functioning of different immune cells, leading to infections and neutropenia (24, 31). For instance, ibrutinib inhibits the Bruton’s tyrosine kinase (BTK), which regulates granulocyte and monocyte function, DC maturation and activation, and B-cell development (31–33).

The precise mode of action of immunomodulatory imide drugs (IMiDs) remains unclear and current hypotheses are mainly based on *in vitro* studies. The immune modulation of IMiDs has been linked to both the innate and adaptive immune responses, including CD4+ and CD8+ T-cell co-stimulation, NK-cell activation, regulatory T-cell (T_reg_) suppression, cytokine production, neutropenia, and increased antibody-dependent cellular cytotoxicity (1, 34). Finally, several drugs with a systemic mode of action not directly linked with the immune system have been used to treat patients with HMs and have adverse events associated with immune system dysfunction. For instance, cytotoxic conventional chemotherapeutics have been shown to affect both the innate and adaptive immune systems by targeting DC, T_reg_, NK cells, cytokine production, and neutrophil and macrophage activity (35). Therefore, physicians need to be aware of the likely immunodeficiency resulting from the combined use of these agents, which affect the correct functioning of multiple immune cell types.

### 1.3 Secondary antibody deficiency (SAD), neutropenia, lymphocytopenia, and hypogammaglobulinemia

Various sub-types of SID have been described based on the components of both the innate and adaptive immune systems that are missing and/or are impaired/malfunctioning (4). For instance, neutropenia, loss of skin and mucosal barrier function, as well as reduced phagocytosis and cytotoxicity are examples of SID related to the innate immune response (4). On the other hand, compromised antibody function and production, and impaired T cells are examples of SID related to the adaptive immune response (4). In an increasing number of cases, defects in T, B, and NK cells may be present at the same time resulting in a combined immunodeficiency (CID) (15).

In this systematic literature review, we will focus on SAD, neutropenia, and lymphocytopenia or diminished lymphocyte function. SAD is defined as a reduction in serum immunoglobulin (Ig) concentration and/or diminished Ig function/quality (3), with hypogammaglobulinemia specifically referring to the aspect of reduction in serum Ig concentration rather than loss of functionality (36). Several cut-offs for hypogammaglobulinemia are used in the literature, suggesting a potential lack of consistency across studies (37–40). The authors agree with the recent expert consensus review published in *Blood Reviews*, where mild (4–6 g/L) and severe (<4 g/L) definitions of hypogammaglobulinaemia are suggested (41).

Neutropenia is a reduction in the absolute number of neutrophils circulating in the blood, graded per the National Cancer Institute (NCI) Common Terminology Criteria for Adverse Events (CTCAE), version 5.0 as grade 1, less than the lower limit of normal–1,500 per mm^3^; grade 2, 1,499–1,000 per mm^3^; grade 3, 999–500 per mm^3^; grade 4, <500 per mm^3^ (42, 43). Lymphocytopenia is a reduction in the total lymphocyte count (i.e., T cells, B cells, and NK cells) graded per NCI-CTAE, version 5.0 as grade 1, less than the lower limit of normal–800 per mm^3^; grade 2, 799–500 per mm^3^; grade 3, 499–200 per mm^3^; grade 4, <200 per mm^3^ or a decreased function of these cells (42–44). However, different institutions may use slightly different reference ranges to determine grading. Lymphocytopenia might not be an ideal marker of a dysfunctional immune system in patients with CLL due to lymphocytosis; however, it could be useful in patients with NHL and MM to define the risk of infections (44–46).

### 1.4 Unmet needs

Epidemiological data on the prevalence of infections and infection-related mortality in patients with HMs suggest infections may account for up to 50% of deaths in CLL, and up to 22% and 33% of deaths in MM and NHL, respectively (7, 47–49). However, it is difficult to confirm whether these infections are linked to hypogammaglobulinemia, impacts on other immune components, comorbidities, or a combination thereof. Furthermore, data are lacking regarding differences in rates of hypogammaglobulinemia and hypogammaglobulinemia-related infections across HMs and across classes of drugs, rates of lymphocytopenia and related infections, and types of infections across HMs. The lack of data may result in a lesser awareness of the issue of hypogammaglobulinemia and infections within this population and therefore a lower uptake in assessment and management strategies for SID in HMs (**Table 1**).

**Table 1:** Current knowledge gaps in the management of SID.

Lack of data	
•Differences in rates of hypogammaglobulinemia-related infections •Rates of hypogammaglobulinemia and infections across classes of drugs (e.g., B-lineage monoclonal antibody, CAR T therapies, etc.) •Types of infections across HMs •Development of improved markers of cellular immunodeficiency
Lack of protocol-based approaches	
•Testing and monitoring Ig levels in patients with HMs (e.g., when; how often) •Testing lymphocyte count before and during therapy as first step in identifying CID •Determining the functional status of the immune system (e.g., test immunization to assess the response to polysaccharide and polypeptide vaccine challenge)
Lack of awareness	
•The impact of cancer agents in developing SID •The risk of death due to SID-related infections

CAR T, chimeric antigen receptor T-cell; CID, combined immunodeficiency; HM, hematological malignancy; Ig, immunoglobulin; SID, secondary immunodeficiency.

### 1.5 Scope of the systematic literature review

In this systematic literature review, we aim to provide insights into the cancer agents used to treat HMs that are associated with SID, including differences in incidence of SID and infections among patients undergoing systemic treatment for CLL, MM, and NHL.

## 2 Methods

This review is reported in accordance with PRISMA guidelines on reporting reviews of the literature. On March 16^th^, 2022, a systematic literature search was performed from the PubMed database, searching for studies that mentioned in the title and/or abstract the following categories of drugs (licensed to treat CLL, MM, or NHL in the EU and US) divided per class of drug (**Table 2**): monoclonal antibodies, CAR T therapies, proteasome inhibitors, kinase inhibitors, IMiDs, corticosteroids, antimetabolites, anti-tumor antibiotics, alkylating agents, Bcl-2 antagonism through Bcl-2 homology 3 (BH3) mimetic, histone deacetylase (HDAC) inhibitors, vinca alkaloids, or selective inhibitors of nuclear export (SINE). In addition, the search strings included the MeSH terms for three types of HMs that are more indolent than others, in which SID is known to be a current unresolved challenge, and for which sufficient studies were expected to be found in order to carry out the analysis: CLL, or MM, or NHL. Finally, the following studies were included: interventional, or observational, or retrospective, or cohort, or meta-analysis, or prospective, or database, or multicenter, or case-control. Further inclusion criteria were applied to identify articles written in English, including humans, labeled as clinical trials in PubMed, and published between 2011 and 2022. Based on agreement among the authors, this period reflects the rapid evolution of the treatment landscape over the last decade.

**Table 2:** Selected drugs used for the search criteria in PubMed.

Classes of drugs	Agents
Alkylating agents	Bendamustine, chlorambucil, cisplatin, cyclophosphamide, ifosfamide, and melphalan
Antimetabolites	Cladribine, cytarabine, fludarabine, methotrexate, nelarabine, pentostatin, and pralatrexate
Anti-tumor antibiotics	Doxorubicin and pixantrone
BH3 mimetic	Venetoclax
CAR T therapies	Axicabtagene ciloleucel, brexucabtagene autoleucel, idecabtagene vicleucel, and tisagenlecleucel
Corticosteroids	Prednisone and dexamethasone
HDAC inhibitors	Panobinostat and vorinostat
IMiDs	Lenalidomide, pomalidomide, and thalidomide
Kinase inhibitors	Acalabrutinib, duvelisib, ibrutinib, and idelalisib
Monoclonal antibodies	Alemtuzumab, belantamab mafodotin, brentuximab vedotin, daratumumab, elotuzumab, isatuximab, obinutuzumab, ofatumumab, and rituximab
Proteasome inhibitors	Bortezomib, carfilzomib, and ixazomib
SINE	Selinexor
Vinca alkaloids	Vincristine

BH3, Bcl-2 antagonism through Bcl-2 homology 3; CAR T, chimeric antigen receptor T-cell; HDAC, histone deacetylase; IMiDs, immunomodulatory imide drugs; SINE, selective inhibitors of nuclear export.

This initial search resulted in 738 publications, which were then further refined to include phase III, phase IV and observational studies only, excluding phase I and phase II studies to avoid considering doses or settings that might not reflect the approved labels and are more likely to have fewer patients enrolled compared with phase III and phase IV studies. We obtained 243 publications in total (**Supplementary Figure 1**) that were then screened for relevance by type of HM, drug regimen, number of patients, year of publication, and class of drug. The screening was performed in parallel to minimize the risk of bias. Double counting was avoided by using the numerical identifier unique to each article and the Excel functionality called ‘distinct count’. In order to be included in this systematic literature review, a publication had to report at least one of the following details related to adverse events (defined per the CTCAE): percentages of patients with any grade or grade ≥3 infections; percentages of patients with any grade or grade ≥3 neutropenia; and percentages of patients with hypogammaglobulinemia. These types of infections were selected as they were the most frequently reported and comparable across all studies. In addition, studies that reported grade 1 and/or grade 2 adverse events only were excluded because of incompatibility with the any grade or grade ≥3 events criteria used in our paper.

Of the 243 studies evaluated, 89 were considered relevant. From the relevant publications, the percentages of patients with lymphocytopenia (composition of lymphocytopenia was not specified) and specific types of infection (fungal, viral, bacterial, lower respiratory tract infection [LRTI], upper respiratory tract infection [URTI], sinusitis, nasopharyngitis, respiratory, bronchitis, pneumonia, urinary tract infection [UTI], skin, gastrointestinal [GI], *Candida*, and sepsis) were collected if available. Of note, not all studies reported values for both any grade and grade ≥3 events; this has led to the situation where in some categories, individual studies only reporting grade ≥3 results reported higher levels of grade ≥3 events than other studies did for any grade events, leading to the average of grade ≥3 events being higher than the average for any grade events. These instances are highlighted in the analysis for clarity.

### 2.1 Types of infection analyses

Further analyses were performed on sinopulmonary bacterial infections and the types of infections that were most reported in the studies evaluated as part of the systematic literature review. The mean percentage of patients with the following types of infections were collected for these analyses: fungal, viral, bacterial, bacteremia, staphylococcal bacteremia, varicella-zoster virus (VZV) reactivation, LRTI, URTI, sinusitis, nasopharyngitis, respiratory, bronchitis, pneumonia, lung, UTI, skin, GI, herpes simplex virus, *Candida* only, and sepsis. Due to some of these descriptors overlapping (e.g., respiratory, lung, LRTI, and URTI), we categorized herpes simplex virus and VZV reactivation within the herpes group viral subtype; sinusitis and nasopharyngitis within the URTI subtype; bacteremia and staphylococcal bacteremia within the bacterial subtype; and lung with the respiratory subtype. Sinopulmonary bacterial infections were calculated by including LRTI, URTI, sinusitis, nasopharyngitis, bronchitis, and/or pneumonia.

## 3 Results

### 3.1 Infection and neutropenia rates in patients with CLL, MM, and NHL

Of the 89 relevant publications, 17 included patients with CLL (50–66), 38 with MM (67–99), and 34 with NHL (100–134) (**Table 3**). The mean proportion of patients who had any grade or grade ≥3 infections was 51.3% and 19.8% in CLL, 35.9% and 16.3% in MM, and 31.1% and 11.3% in NHL, respectively (**Table 3**). The mean percentage of patients who had any grade neutropenia was 36.3% in CLL, 36.4% in MM, and 35.4% in NHL. The mean percentage of patients with grade ≥3 neutropenia was 29.8% in patients with CLL, 23.2% in patients with MM, and 38.7% in patients with NHL.

**Table 3:** Percentages of patients with CLL, MM, and NHL who had infections (any grade and grade ≥3), neutropenia (any grade or grade ≥3), or hypogammaglobulinemia.

Malignancies		Any grade neutropenia^*^		Grade ≥3 neutropenia^*^		Any grade infections^*^		Grade ≥3 infections^*^		Hypogamma^*^
Studies (n)		Mean	Range	Mean	Range	Mean	Range	Mean	Range	Range
CLL	17	36.3	9.4–64.0	29.8	3.0–60.0	51.3	14.4–69.1	19.8	6.4–39.0	0.0–15.3
MM	38	36.4	9.8–85.5	23.2	2.0–80.0	35.9	0.0–68.0	16.3	0.0–50.2	–
NHL	34	35.4	3.2–87.5	38.7	0.0–100.0	31.1	4.0–81.0	11.3	0.9–38.0	5.9
Total	89	36.0	3.2–87.5	29.6	0.0–100.0	36.7	0.0–81.0	15.9	0.0–50.2	0.0–15.3

*The reporting criteria for time to adverse events differed across studies.

Neutropenia grades: grade 1, less than the lower limit of normal–1,500 per mm3; grade 2, 1,499–1,000 per mm^3^; grade 3, 999–500 per mm^3^; grade 4, <500 per mm^3^; grade 5, death.

Infection grades: grade 1, –; grade 2, localized, local intervention indicated; grade 3, IV antibiotic, antifungal, or antiviral intervention indicated, interventional radiology or operative intervention indicated; grade 4, life-threatening consequences e.g., septic shock, hypotension, acidosis, or necrosis; grade 5, death.

CLL, chronic lymphocytic leukemia; hypogamma, hypogammaglobulinemia; IV, intravenous; MM, multiple myeloma; NHL, non-Hodgkin lymphoma; –, not reported.

In addition, rates of any grade and grade ≥3 infections, neutropenia, and hypogammaglobulinemia were divided into two timeframe groups to reflect changes in the treatment landscape, 2011–2016 and 2017–2022 (**Supplementary Table 1**). The rates of grade ≥3 infections were higher in the 2017–2022 group versus the 2011–2016 group across CLL, MM, and NHL.

**Figure 1:**
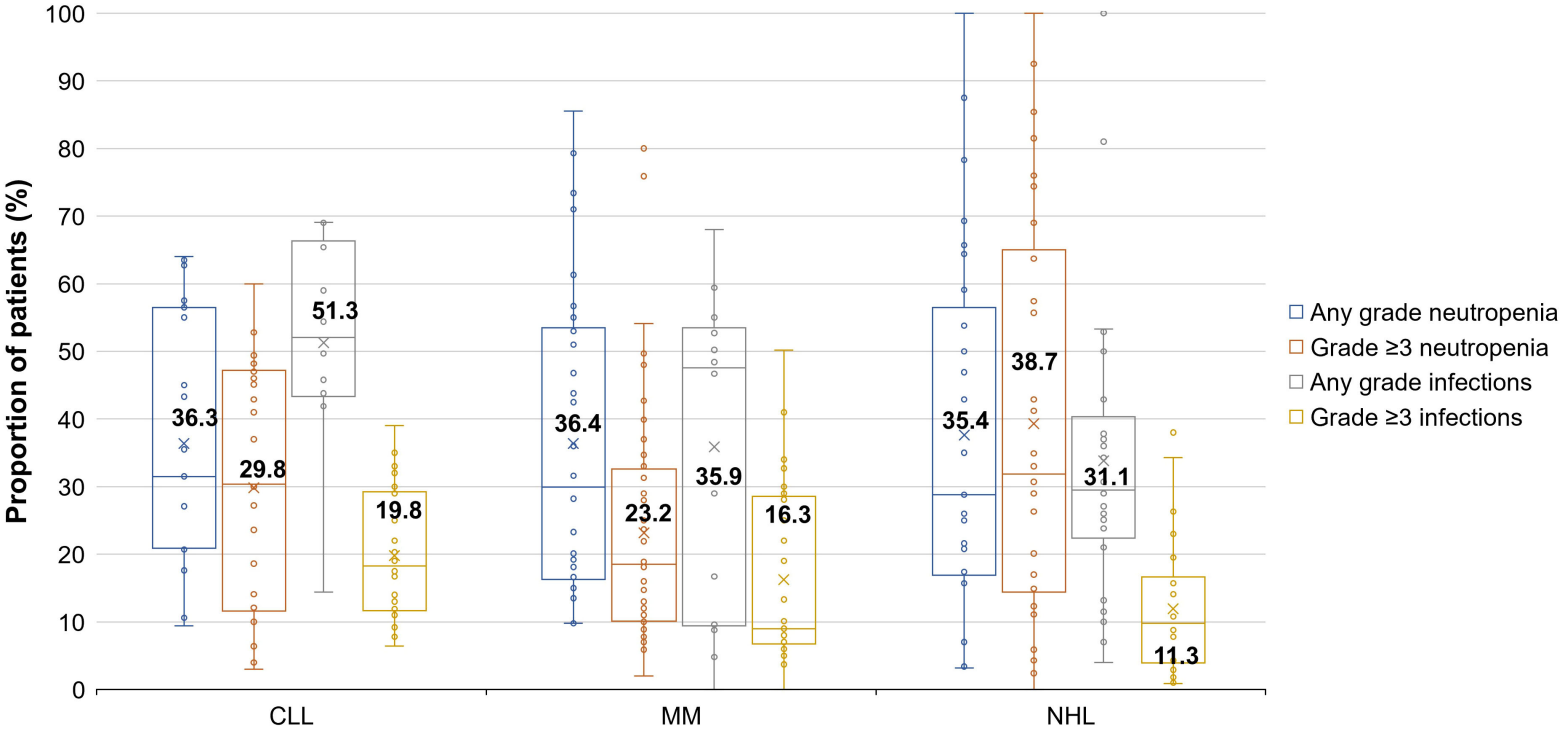
Proportions of patients with CLL, MM, and NHL who had infections (any grade and grade ≥3) or neutropenia (any grade or grade ≥3). Each box displays data distribution through their quartile (i.e., upper quartile, median, and lower quartile), with the bars representing the variability outside the upper and lower quartile (i.e., upper extreme and lower extreme). A dot outside the bars represents an outlier. The x symbols and corresponding data label represent the mean values for each data set. Not all studies reported values for both any grade and grade ≥3 events; this has led to the situation where in some categories, individual studies reported higher levels of grade ≥3 events than other studies did for any grade events, leading to the average of grade ≥3 events being higher than the average for any grade events Neutropenia grades: grade 1, less than the lower limit of normal–1,500 per mm^3^; grade 2, 1,499–1,000 per mm^3^; grade 3, 999–500 per mm^3^; grade 4, <500 per mm^3^; grade 5, death. Infection grades: grade 1, –; grade 2, localized, local intervention indicated; grade 3, IV antibiotic, antifungal, or antiviral intervention indicated, interventional radiology or operative intervention indicated; grade 4, life-threatening consequences e.g., septic shock, hypotension, acidosis, or necrosis; grade 5, death. CLL, chronic lymphocytic leukemia; IV, intravenous; MM, multiple myeloma; NHL, non-Hodgkin lymphoma.

The high variability across studies resulted in extremely wide ranges of neutropenia and infection rates. For this reason, box and whisker plots (**Figure 1**) were created to locate each percentage of patients within the ranges. As shown in the box and whisker plots, patients with CLL seem to be more susceptible to any grade and grade ≥3 infections than patients with MM and NHL.

### 3.2 Drug class-related analyses

Drug class-related analyses were performed and included all studies where a B-lineage monoclonal antibody, a proteasome inhibitor, a kinase inhibitor, or immunomodulatory drugs were used either as monotherapy or in combination with different classes of drugs as a doublet or triplet regimen (**Table 4**). The sum of the number of studies for monotherapy and doublet/triplet regimens in **Table 4** may be higher than the total number of studies reported in **Table 3** as some studies may have both monotherapy and doublet/triplet arms, therefore may have been counted twice. Only one study on the use of CAR T therapies in patients with NHL resulted from the systematic literature review; the proportion of patients with any grade infection was 29.5% (104).

**Table 4:** Percentages of patients with CLL, MM, and NHL treated with different classes of drugs as monotherapy or in combination with other therapies who had infections (any grade and grade ≥3), neutropenia (any grade or grade ≥3), or hypogammaglobulinemia.

Malignancies		Any grade neutropenia^*^		Grade ≥3 neutropenia^*^		Any grade infections^*^		Grade ≥3 infections^*^		Hypogamma^*^
Studies (n)		Mean	Range	Mean	Range	Mean	Range	Mean	Range	Range
B-lineage monoclonal antibodies (as doublet, triplet etc.)										
CLL	14	44.9	20.9–64.0	35.2	3.0–60.0	50.2	14.4–69.1	20.6	6.4–39.0	15.3
MM	5	27.5	9.8–61.3	20.5	5.9–54.1	–	–	41.0	41.0	–
NHL	18	51.5	17.4–87.5	50.8	11.1–92.5†	33.0	10.0–53.3	8.5	0.9–23.0	–
Total	37	44.1	9.8–87.5	38.9	3.0–92.5	40.6	10.0–69.1	16.7	0.9–41.0	15.3
B-lineage monoclonal antibodies (as monotherapy)										
CLL	3	24.4	20.9–27.8	15.3	7.0–23.6	–	–	19.7	11.0–35	–
MM	2	16.4	13.5–19.2	10.4	7.8–13.1	50.2	50.2	13.3	13.3	–
NHL	7	14.1	3.4–22.0	8.5	2.4–14.9	23.0	7.0–36.1	3.6	2.0–4.4	5.9
Total	12	16.9	3.4–27.8	10.7	2.4–23.6	27.5	7.0–50.2	11.9	2.0–35	5.9
Proteasome inhibitors (as doublets, triplets etc.)										
MM	18	34.3	14.0–73.4	21.1	7.0–42.7	25.9	9.6–48.4	8.5	3.8–13.5	–
NHL	2	32.2	17.4–46.9	23.0	11.1–34.9	53.3	53.3	6.9	2.9–10.8	–
Total	20	33.9	14.0–73.4	21.4	7.0–42.7	31.4	9.6–53.3	8.1	2.9–13.5	–
Proteasome inhibitors (as monotherapy)										
MM	9	50.0	28.2–79.3	13.2	2.0–25.0	8.8	8.8	16.9	3.7–30.0†	–
NHL	3	25.5	25.0–26.0	11.5	5.9–17.0	–	–	–	–	–
Total	12	40.2	25.0–79.3	12.7	2.0–25.0	8.8	8.8	16.9	3.7–30.0	–
Kinase inhibitors (as doublets, triplets etc.)										
CLL	5	39.0	20.9–64.0	30.4	6.4–60.0	69.1	69.0–69.1	29.9	20.8–39.0	15.3
NHL	2	31.9	20.8–42.9	24.3	15.6–33	42.9	42.9	38.0	38.0	–
Total	7	37.0	20.8–64.0	29.4	6.4–60.0	60.3	42.9–69.1	32.6	20.8–39.0	15.3
Kinase inhibitors (as monotherapy)										
CLL	3	13.6	9.4–20.7	8.7	4.1–12.1	57.6	49.7–65.4	14.0	14.0	0.0
NHL	1	16.0	16.0	13.0	13.0	–	–	–	–	–
Total	4	14.2	9.4–20.7	9.8	4.1–13.0	57.6	49.7–65.4	14.0	14.0	0.0
IMiDs (as doublets, triplets etc.)										
MM	12	25.9	15.0–43.8	19.2	8.0–37.0	44.8	16.7–59.4	12.0	6.0–29.0	–
NHL	1	36.1	22.1–50.0	–	–	–	–	–	–	–
Total	13	28.8	15.0–50.0	19.2	8.0–37.0	44.8	16.7–59.4	12.0	6.0–29.0	–
IMiDs (lenalidomide and thalidomide as monotherapy)										
MM	5	51.3	31.6–71.0	29.7	13.0–43.0	–	–	21.1	5.0–50.2	–
NHL	2	15.7	15.7	20.1	20.1	29.0	29.0	10.8	10.8	–
Total	7	39.4	15.7–71.0	27.3	13.0–43.0	29.0	29.0	19.0	5.0–50.2	–
Non-specific agents (immunomodulators, antimetabolites, anti-tumor antibiotics, alkylating agents, mitotic inhibitors)										
CLL	1	17.6	17.6	14.1	14.1	45.8	45.8	11.9	11.9	–
MM	2	85.5	85.5	78.0	75.9–80.0	4.8	4.8	11.5	4.0–19.0	–
NHL	5	48.2	28.8–65.7	46.6	26.3–76.0†	19.4	4.0–34.3	19.6	8.8–34.3	–
Total	8	49.5	17.6–85.5	50.9	14.1–80.0	21.4	4.0–45.8	15.6	4.0–34.3	–

*The reporting criteria for time to adverse events differed across studies.

†Not all studies reported values for both any grade and grade ≥3 events; this has led to the situation where in some categories, individual studies reported higher levels of grade ≥3 events than other studies did for any grade events, leading to the average of grade ≥3 events being higher than the average for any grade events.

Neutropenia grades: grade 1, less than the lower limit of normal–1,500 per mm^3^; grade 2, 1,499–1,000 per mm^3^; grade 3, 999–500 per mm^3^; grade 4, <500 per mm^3^; grade 5, death.

Infection grades: grade 1, –; grade 2, localized, local intervention indicated; grade 3, IV antibiotic, antifungal, or antiviral intervention indicated, interventional radiology or operative intervention indicated; grade 4, life-threatening consequences e.g., septic shock, hypotension, acidosis, or necrosis; grade 5, death.

#### 3.2.1 B-lineage monoclonal antibodies

B-lineage monoclonal antibodies (anti-CD20: rituximab, ofatumumab, and obinutuzumab; anti-CD38: isatuximab and daratumumab; anti-CD30: brentuximab vedotin; anti-CD52: alemtuzumab; anti-CD269: belantamab mafodotin; or anti-CD319: elotuzumab) were used in three studies in CLL (54, 59, 66), two in MM (68, 91), and seven in NHL (110, 113, 122, 124, 127, 128, 134) as monotherapy. In addition, 14 studies in CLL (50–58, 61–65), five studies in MM (87, 90, 92, 97, 99), and 18 studies in NHL (105–107, 111, 112, 114, 115, 117–121, 124, 127, 130–133) reported use of B-lineage monoclonal antibodies in combination with other agents (**Table 4**).

The mean proportion of patients treated with a B-lineage monoclonal antibody as monotherapy who had any grade infections was 50.2% in MM and 23.0% in NHL. In patients with CLL, no publications reported the rate of any grade infections when a B-lineage monoclonal antibody was used as monotherapy. Grade ≥3 infections were similar in patients with CLL and NHL regardless of using a B-lineage monoclonal antibody as monotherapy (19.7% in CLL and 3.6% in NHL) or in combination with other agents (20.6% in CLL and 8.5% in NHL); however, grade ≥3 infections were numerically lower in patients with MM treated with a B-lineage monoclonal antibody as monotherapy (13.3%) compared with patients treated with doublet/triplet regimen that included a B-lineage monoclonal antibody (41.0%; **Table 4**). The mean proportion of patients who reported any grade and grade ≥3 neutropenia was often numerically lower in patients treated with a B-lineage monoclonal antibody as monotherapy compared with patients treated with doublet/triplet regimen in patients with CLL, MM, and NHL (**Table 4**).

#### 3.2.2 Proteasome inhibitors

Proteasome inhibitors used as monotherapy were reported in nine studies in MM (69, 70, 80, 81, 83, 94, 95, 98, 135) and three in NHL (109, 123, 126). In addition, 18 studies in MM (71, 76–78, 82, 84–87, 89, 90, 93, 96–99, 136) and two studies in NHL (107, 124) reported the use in combination with other agents (**Table 4**). No data were reported on the use of proteasome in patients with CLL. Data on infections were not reported in patients treated with proteasome inhibitor monotherapy in patients with NHL. In patients with MM, any grade infections were numerically lower in patients treated with a proteasome inhibitor as monotherapy compared with doublet/triplet regimen; however, grade ≥3 infections were numerically higher in patients who received mono versus combination therapy (**Table 4**). The mean proportion of patients with grade ≥3 neutropenia was lower in patients treated with proteasome inhibitor monotherapy compared with patients treated with doublet/triplet regimen (**Table 4**). However, fewer studies reported the use of a proteasome inhibitor as monotherapy compared with combination therapy, which might have skewed the results.

#### 3.2.3 Kinase inhibitors

Kinase inhibitors were reported in three studies in CLL (51, 60, 62) and one in NHL (123) when used as monotherapy, and in five studies in CLL (50, 51, 56, 61, 62) and two studies in NHL (100, 127) in combination with other therapies. The mean proportion of patients treated with a kinase inhibitor as monotherapy who had any grade and grade ≥3 infections was 57.6% and 14.0% in CLL, respectively. Data on any grade and grade ≥3 infections were not reported in patients with MM or NHL treated with a kinase inhibitor. The mean proportion of patients treated with a kinase inhibitor in combination with other therapies who had any grade and grade ≥3 infections was 69.1% and 29.9% in CLL, and 42.9% and 38.0% in NHL, respectively. The mean proportion of patients with any grade/grade ≥3 infections and neutropenia was numerically lower in patients with CLL treated with a kinase inhibitor as monotherapy versus combination therapy (any grade and grade ≥3 infections: 57.6% and 14.0% versus 69.1% and 29.9%, respectively; any grade and grade ≥3 neutropenia: 13.6% and 8.7% versus 39.0% and 30.4%, respectively, **Table 4**).

#### 3.2.4 IMiDs

IMiDs were used as monotherapy in five studies in MM (69, 73, 75, 79, 135) and two in NHL (108, 125). In addition, IMiDs were used in combination with other therapies in 12 studies in MM (67, 71–74, 77, 79, 84, 89, 92, 136, 137) and one study in NHL (111). No studies reported data on IMiDs in CLL. In patients with MM treated with monotherapy, only grade ≥3 infections were reported, and the mean rate was 21.1%. The mean proportion of patients treated with monotherapy who had any grade and grade ≥3 neutropenia was 51.3% and 29.7% in MM, respectively. In patients with NHL, the mean proportion of patients with any grade and grade ≥3 infections was 29.0% and 10.8%, respectively, and the mean proportion of patients who had any grade and grade ≥3 neutropenia was 15.7% and 20.1%, respectively. When used as monotherapy in patients with MM, IMiDs led to a numerically higher rate of grade ≥3 infections compared with IMiDs used in combination with other therapies (**Table 4**).

#### 3.2.5 Non-specific agents

Additional analyses for non-specific agents were performed and included only corticosteroids, antimetabolites, anti-tumor antibiotics, alkylating agents, and mitotic inhibitors regardless of whether these were monotherapy or combination regimen (**Table 4**). These analyses did not include B-lineage monoclonal antibodies, tyrosine kinase inhibitors, proteasome inhibitors, or IMiDs. Non-specific agents were used in eight studies, one in CLL (65), two in MM (79, 82), and five in NHL (102, 115, 116, 119, 129). In CLL, the proportion of patients with any grade and grade ≥3 infections was 45.8% and 11.9%, respectively. The mean percentage for any grade and grade ≥3 infections was 4.8% and 11.5% in patients with MM, respectively (this is due to individual studies reporting only grade ≥3 results that were higher than other studies reported for any grade events), and 19.4% and 8.8% in patients with NHL, respectively. The mean proportion of patients who had any grade and grade ≥3 neutropenia are shown in **Table 4**.

### 3.3 Specific drug analyses

Analyses were performed to estimate the ranges of patients with infections, neutropenia or hypogammaglobulinemia associated with specific drug use (**Table 5**). These analyses included the use of drugs as monotherapy or in combination with other agents. Only the drugs with the highest number of studies in each class of drug were selected for these analyses.

**Table 5:** Percentages of patients with CLL, MM, and NHL treated with rituximab, bortezomib, ibrutinib, lenalidomide, or dexamethasone as monotherapy or in combination with other therapies who had infections (any grade and grade ≥3), neutropenia (any grade or grade ≥3), or hypogammaglobulinemia.

Malignancies	Studies (n)	Any grade neutropenia*		Grade ≥3 neutropenia*		Any grade infections*		Grade ≥3 infections*		Hypogamma*
		Mean	Range	Mean	Range	Mean	Range	Mean	Range	Range
Rituximab (as doublets, triplets etc.)										
CLL	7	46.6	20.9–64.0	36.2	3.0–60.0	64.0	59.0–69.0	23.8	6.4–39.0	–
NHL	17	53.0	17.4–87.5	51.9	11.1–92.5	33.0	10.0–53.3	8.5	0.9–23.0	–
Total	24	51.6	17.4–87.5	46.7	3.0–92.5	38.7	10.0–69.0	15.8	0.9–39.0	–
Rituximab (as monotherapy)										
CLL	2	20.9	20.9	7.0	7.0	–	–	15.0	11.0–19.0	–
NHL	7	14.1	3.4–22.0	8.5	2.4–14.9	23.0	7.0–36.1	3.6	2.0–4.4	5.9
Total	9	15.2	3.4–22.0	8.2	2.4–14.9	23.0	7.0–36.1	8.1	2.0–19	5.9
Bortezomib (as doublets, triplets etc.)										
MM	14	36.2	18.1–73.4	24.8	9.2–42.7	25.9	9.6–48.4	8.5	3.8–13.5	–
NHL	1	17.4	17.4	11.1	11.1	53.3	53.3	10.8	10.8	–
Total	15	33.1	17.4–73.4	23.7	9.2–42.7	31.4	9.6–53.3	8.8	3.8–13.5	–
Bortezomib (as monotherapy)										
MM	7	42.5	42.5	10.6	2.0–25.0	8.8	8.8	16.9	3.7–30.0†	–
NHL	2	25.0	25.0	5.9	5.9	–	–	–	–	–
Total	9	33.8	25.0–42.5	9.6	2.0–25.0	8.8	8.8	16.9	3.7–30.0	–
Ibrutinib (as doublets, triplets etc.)										
CLL	2	39.4	35.5–43.3	27.8	18.6–37.0	–	–	–	–	15.3
NHL	1	42.9	42.9	33.0	33.0	42.9	42.9	38.0	38.0	–
Total	3	40.6	35.5–43.3	29.5	18.6–37.0	42.9	42.9	38.0	38.0	15.3
Ibrutinib (as monotherapy)										
CLL	2	15.1	9.4–20.7	8.1	4.1–12.1	49.7	49.7	–	–	0.0
NHL	1	16.0	16.0	13.0	13.0	–	–	–	–	–
Total	3	15.4	9.4–20.7	9.7	4.1–13.0	49.7	49.7	–	–	0.0
Lenalidomide (as doublets, triplets etc.)										
MM	10	32.5	15.0–61.3	23.0	8.0–54.1	52.5	46.7–59.4	19.5	6.0–41.0	–
NHL	1	36.1	22.1–50.0	–	–	–	–	–	–	–
Total	11	33.4	15.0–61.3	23.0	8.0–54.1	52.5	29.0–59.4	19.5	6.0–41.0	–
Lenalidomide (as monotherapy)										
MM	3	71.0	71.0	29.7	13.0–43.0	–	–	25.7	5.0–50.2	–
NHL	2	15.7	15.7	20.1	20.1†	29.0	29.0	10.8	10.8	–
Total	5	43.4	15.7–71.0	27.3	13.0–43.0	29.0	29.0	22.0	5.0–50.2	–
Dexamethasone (as doublets, triplets etc.)										
MM	21	34.2	9.8–73.4	22.5	5.9–54.1	46.8	9.6–68.0	18.4	6.0–41.0	–
NHL	3	10.8	3.2–22.1	34.8	0.0–100.0†	51.6	36.0–81.0	12.7	11.3–14.1	–
Total	24	30.5	3.2–73.4	23.3	0.0–100.0	48.6	9.6–81.0	17.7	6.0–41.0	–
Dexamethasone (as monotherapy)										
MM	1	20.1	20.1	16	16	52.7	52.7	32.7	32.7	–

*The reporting criteria for time to adverse events differed across studies.

†Not all studies reported values for both any grade and grade ≥3 events; this has led to the situation where in some categories, individual studies reported higher levels of grade ≥3 events than other studies did for any grade events, leading to the average of grade ≥3 events being higher than the average for any grade events.

Neutropenia grades: grade 1, less than the lower limit of normal–1,500 per mm^3^; grade 2, 1,499–1,000 per mm^3^; grade 3, 999–500 per mm^3^; grade 4, <500 per mm^3^; grade 5, death.

Infection grades: grade 1, –; grade 2, localized, local intervention indicated; grade 3, IV antibiotic, antifungal, or antiviral intervention indicated, interventional radiology or operative intervention indicated; grade 4, life-threatening consequences e.g., septic shock, hypotension, acidosis, or necrosis; grade 5, death.

CLL, chronic lymphocytic leukemia; hypogamma, hypogammaglobulinemia; IV, intravenous; MM, multiple myeloma; NHL, non-Hodgkin lymphoma; –, not reported.

#### 3.3.1 Rituximab

In the nine studies that evaluated the anti-CD20 agent rituximab as monotherapy [two in CLL (54, 66) and seven in NHL (110, 113, 115, 122, 124, 127, 128)], the mean percentage for any grade and grade ≥3 infections was 23% and 3.6% in patients with NHL, respectively (**Table 5**). Only grade ≥3 infections and neutropenia were reported in patients with CLL, and the rates were 15.0% and 7.0%, respectively. The mean percentage for any grade and grade ≥3 neutropenia was 14.1% and 8.5% in patients with NHL, respectively.

Twenty-four studies evaluated rituximab [seven in CLL (50, 53, 54, 56–58, 63) and 17 in NHL (105–107, 111, 112, 114, 115, 117, 118, 120, 121, 124, 127, 130–133)] in combination with other therapies (**Table 5**).When rituximab was used as monotherapy, the rates of any grade and ≥3 infections and neutropenia were numerically lower across CLL, MM, and NHL compared with rituximab used in combination with other therapies (**Table 5**).

#### 3.3.2 Bortezomib

Bortezomib was evaluated in nine studies as monotherapy [seven in MM (69, 81, 83, 94, 95, 98, 135) and two in NHL (109, 126)] and in 15 studies (14 in MM (71, 74, 76–78, 82, 84–86, 89, 90, 96, 98, 136) and one in NHL) in combination with other therapies. No studies reported the use of bortezomib in patients with CLL. When used as monotherapy in patients with MM, bortezomib led to a higher rate of grade ≥3 infections compared with its use in combination with other therapies (**Table 5**).

#### 3.3.3 Ibrutinib

Three studies evaluated the use of ibrutinib as monotherapy, two in CLL (60, 62) and one in NHL (123), and three studies in combination with other therapies [two in CLL (61, 62) and one in NHL (100)]. In patients treated with ibrutinib monotherapy, only the mean percentage for any grade infections was reported and only in patients with CLL (49.7%). When used in combination with other therapies, only the mean percentage for any grade and grade ≥3 infections was reported in patients with NHL, and the rates were 42.9% and 38.0%, respectively (**Table 5**).

Both any grade and grade ≥3 neutropenia were numerically lower in both patients with CLL and NHL when treated with ibrutinib monotherapy compared with ibrutinib included in doublet/triplet regimen (**Table 5**).

#### 3.3.4 Lenalidomide

Lenalidomide was used as monotherapy in five studies [three in MM (69, 75, 79) and two in NHL (108, 125)], and in combination with other therapies in 11 studies [10 in MM (67, 71–74, 77, 79, 92, 93, 137) and one in NHL (111)]. When used as monotherapy, the mean percentage for grade ≥3 infections was 25.7% in patients with MM, and the mean percentage for any grade and grade ≥3 infections was 29% and 10.8% in patients with NHL, respectively. Data on any grade infections were not reported in patients with MM treated with lenalidomide monotherapy (**Table 5**). The mean percentage for any grade and grade ≥3 neutropenia was 71% and 29.7% in patients with MM, respectively, and 15.7% and 20.1% in patients with NHL, respectively. Data for combination with other therapies, are shown in **Table 5**.

#### 3.3.5 Dexamethasone

The use of dexamethasone as monotherapy was reported in only one MM study (88). Combination with other therapies was reported in 24 studies, 21 in MM (67, 71–74, 77–79, 82, 84, 86–88, 92, 93, 96, 97, 99, 136–138) and three in NHL (101, 103, 111).

For combination regimens, in which dexamethasone was used with a diverse range of agents, the mean percentage for any grade and grade ≥3 infections was 46.8% and 18.4% in patients with MM, and 51.6% and 12.7% in patients with NHL, respectively. The mean percentage for any grade and grade ≥3 neutropenia was 34.2% and 22.5% in patients with MM, and 10.8% and 34.8% in patients with NHL, respectively.

### 3.4 Infection and neutropenia rates in patients receiving regimen combinations commonly used in clinical practice

The drugs with the highest number of studies in each class of drug were selected for the drug specific analyses. However, in clinical practice, certain specific drug combinations are more commonly used than others, such as those recommended by the European Society for Medical Oncology (ESMO) (139–141).

When assessing these more commonly used combinations, seven studies reported the use of rituximab in combination with cyclophosphamide, doxorubicin, vincristine, and prednisone (R-CHOP) in patients with NHL (106, 107, 114, 120, 130, 131, 133). In this population, the mean percentage for any grade and grade ≥3 infections was 34.9% and 11.3%, respectively; and the mean percentage for any grade and grade ≥3 neutropenia was 67.1% and 64.5%, respectively.

In patients with CLL, the use of chlorambucil in combination with obinutuzumab was reported in five studies (G-Clb); fludarabine, cyclophosphamide, and rituximab (FCR) in three studies; and bendamustine plus rituximab in four studies. None of the selected studies reported data on the use of venetoclax in combination with obinutuzumab. In the G-Clb group, the mean percentage for any grade and grade ≥3 infections was 29.1% and 11.1%, respectively; and the mean percentage for any grade and grade ≥3 neutropenia was 46.5% and 38.4%, respectively (51, 52, 61, 63, 64). In the FCR group, only the mean percentage for grade ≥3 infections and neutropenia was reported: 24.1% and 26.0%, respectively (53, 54, 57). In patients who received bendamustine plus rituximab, the mean percentage for any grade and grade ≥3 infections was 42.2% and 18.0%, respectively; and the mean percentage for any grade and grade ≥3 neutropenia was 59.7% and 43.4%, respectively (54, 56, 58, 63).

In one study that investigated the use of daratumumab in combination with lenalidomide and dexamethasone in patients with MM, the rate for grade ≥3 infections was 41.0% and the rates for any grade and grade ≥3 neutropenia were 61.3% and 54.1%, respectively (92). Only the rate for grade ≥3 neutropenia (39.9%) was reported in patients with MM who received daratumumab in combination with bortezomib, melphalan, and prednisone (90). None of the selected studies reported data on both the use of bortezomib in combination with lenalidomide and dexamethasone (VRd) and the use of daratumumab in combination with bortezomib, thalidomide, and dexamethasone (daraVTD) in patients with MM.

None of these studies reported data on the rates of hypogammaglobulinemia, highlighting the need for further reporting on immunoglobulin G (IgG) levels, especially in regimen combinations including drugs known to have a mode of action likely to impact IgG levels directly, such as B-lineage monoclonal antibodies like daratumumab.

### 3.5 Lymphocytopenia in patients with CLL, MM, and NHL

The rates of lymphocytopenia were reported in a limited number of studies only: one in patients with CLL (64), seven in MM (68, 70, 78, 91, 92, 97, 138), and three in NHL (116, 120, 127) (data not shown). The mean percentage of patients with lymphocytopenia was 1.9% in patients with CLL, 11.9% in MM, and 38.6% in NHL.

### 3.6 Hypogammaglobulinemia and sinopulmonary bacterial infection analyses

Only two of the evaluated studies reported data on hypogammaglobulinemia (**Table 3**). In patients with CLL, one study reported hypogammaglobulinemia in 15.3% of patients who received combination therapy with ublituximab (anti-CD20) and ibrutinib, and in 0.0% of patients who received ibrutinib monotherapy (62). In patients with NHL, one study reported hypogammaglobulinemia in 5.9% of patients who received rituximab maintenance therapy for up to 2 years (122). Neither study had a confirmed definition of what was classed as hypogammaglobulinemia nor was testing reported prior to the initiation of treatment.

Patients with hypogammaglobulinemia commonly present with recurrent bacterial sinopulmonary infections (e.g., otitis, sinusitis, pneumonia, nasopharyngitis), which often are due to encapsulated bacteria such as *S. pneumoniae* (3–5, 26, 142). In this systematic literature review, we classed sinopulmonary bacterial infections to include LRTI, URTI, sinusitis, nasopharyngitis, bronchitis, and/or pneumonia, which were collected from 17 studies in CLL, 38 in MM, and 34 in NHL. However, not all relevant studies reported all types of infections used to calculate the rate of sinopulmonary bacterial infections (e.g., no relevant studies reported data on the percentages of patients with LRTI and sinusitis in MM; as a result, the data for sinopulmonary bacterial infections in patients with MM did not include LRTI and sinusitis values). The mean proportion of patients with sinopulmonary bacterial infections was 7.6%, 14.4%, and 6.3% in patients with CLL, MM, and NHL, respectively. Sinopulmonary bacterial infections were reported in 15.7% (54, 59, 66), 8.5% (68, 91), and 7.8% (110, 113, 115, 122, 124, 127, 128, 134) of patients with CLL, MM, and NHL, respectively, when treated with B-lineage monoclonal antibodies as monotherapy (**Table 6**). In patients who received proteasome inhibitor as monotherapy, sinopulmonary bacterial infections were reported in 7.7% (69, 70, 80, 81, 83, 94, 95, 98, 135) of patients with MM. In patients treated with kinase monotherapy, sinopulmonary bacterial infections were reported in 8.0% of patients with CLL (51, 60, 62). In those patients who received non-specific agents as monotherapy and/or double/triplet regimen, sinopulmonary bacterial infections were reported in 9.9% (102, 115, 116, 119, 129) of patients with NHL.

**Table 6:** Proportions of patients with CLL, MM, and NHL treated with different classes of drugs as monotherapy who had sinopulmonary bacterial infections.

Malignancies	Studies (n)	Mean (%)^*^
B-lineage monoclonal antibodies		
CLL	3	15.7
MM	2	8.5
NHL	7	7.8
Proteasome inhibitors		
MM	9	8.8
Kinase inhibitors		
CLL	3	7.7
Non-specific agents		
NHL	5	9.9

*The reporting criteria for time to adverse events differed across studies.

Neutropenia grades: grade 1, less than the lower limit of normal–1,500 per mm^3^; grade 2, 1,499–1,000 per mm^3^; grade 3, 999–500 per mm^3^; grade 4, <500 per mm^3^; grade 5, death.

Infection grades: grade 1, –; grade 2, localized, local intervention indicated; grade 3, IV antibiotic, antifungal, or antiviral intervention indicated, interventional radiology or operative intervention indicated; grade 4, life-threatening consequences e.g., septic shock, hypotension, acidosis, or necrosis; grade 5, death.

CLL, chronic lymphocytic leukemia; IV, intravenous; MM, multiple myeloma; NHL, non-Hodgkin lymphoma.

In this systematic literature review, the most common types of infections reported in patients with CLL and MM were related to the respiratory system, whereas in patients with NHL they were bacterial infections, pneumonia, and viral infections (data not shown). Other less common types of infections included viral and UTI infections in CLL, viral and skin infections in MM, and UTI and *Candida* infections in NHL (data not shown).

## 4 Discussion

Despite infections related to SID accounting for 22–50% of deaths in patients with HMs (7, 47–49), we still observed a lack of data reported on hypogammaglobulinemia, lymphocytopenia, and consistent infection reporting in phase III, phase IV, and observational studies, suggesting a likely underestimate of hypogammaglobulinemia and cellular immunodeficiency in the development of recurrent and fatal infections in patients with HMs. In addition, there is still a lack of data in the literature regarding differences in rates of hypogammaglobulinemia-related infections, rates of hypogammaglobulinemia and infections across classes of drugs, and types of infections across HMs (**Table 1**).

This level of granularity in reporting rates of specific subtypes of SID and related infections might not be a priority for hematologists and hemato-oncologists with the main focus on treating the malignancy. However, we believe that collecting these data might help highlight trends and possible correlations that could inform changes in the management of HMs and related infections in everyday clinical practice, such as improving supportive care and serving as a stimulus for development of approaches that include the early testing and detection of immunodeficiency alongside prevention and treatment of infection as part of the routine management of these HMs (41). Therefore, we undertook this systematic literature review to provide insight into the cancer treatments associated with SID, including the incidence of infections, neutropenia, and hypogammaglobulinemia among patients undergoing systemic treatment for CLL, MM, and NHL.

In this systematic literature review, the highest proportion of patients with grade ≥3 infections across classes of drugs was 41.0% in patients with MM treated with a B-lineage monoclonal antibody combination; and 29.9% and 38.0% of patients with CLL and NHL treated with a kinase inhibitor combination, respectively. As expected, the incidence of neutropenia did not always correlate with the incidence of infections. Interestingly, the higher rates of grade ≥3 infections in the 2017–2022 group versus the 2011–2016 group across all the selected HMs might be due to numerous factors such as the concomitant use of old and novel therapeutic agents (e.g., B-lineage monoclonal antibodies, tyrosine kinase inhibitors, and proteasome inhibitors) and HM therapies becoming increasingly more potent and correspondingly more immunosuppressive, as well as longer survival and more comorbidities.

As many CAR T therapies are still in phase I or II clinical development (143–145) (and were therefore excluded from this systematic literature review) SID data associated with CAR T therapies is still emerging and not fully represented in this systematic literature review. For example, lisocabtagene maraleucel and ciltacabtagene autoleucel were not included in our analyses because these drugs were not approved in both the US and EU markets by March 16, 2022, and therefore there is a relatively small number of patients treated with these agents. Further work is needed in the rapidly evolving field of CAR T to report data on SID-related and hypogammaglobulinemia-related infections (144, 146).

Notably, the use of monotherapy was mostly associated with a numerically lower risk of infection or neutropenia. For instance, the mean proportion of patients with any grade infections was numerically lower when rituximab was used as monotherapy across patients with CLL, MM, and NHL compared with its use in combination with other agents. The use of ibrutinib as monotherapy led to a numerically lower mean percentage of patients with any grade and grade ≥3 neutropenia versus combination therapies. On the contrary, bortezomib used as monotherapy was associated with a numerically higher mean percentage of patients with grade ≥3 infections and a numerically lower mean percentage of patients with any grade infections; as already mentioned, this is due to individual studies reporting only grade ≥3 results that were higher than other studies did for any grade events. Unfortunately, further analyses to compare anti-CD20 versus anti-CD38 agents could not be undertaken due to sample sizes and mismatched disease cohorts.

The infection spectrum observed in this patient population has some similarities with those observed in primary antibody deficiency (PAD) but also some differences. While sinopulmonary infections are common in HMs and PAD, infection sites that are less common in PAD were also observed in this systematic literature review, such as the urinary tract and skin (with herpes group viral reactivation/infection in particular). The occurrence of viral and fungal, as well as bacterial pathogen groups, speaks to a potential CID phenotype in many patients with HM. The variability in the types of infection across patients with CLL, MM, and NHL might be due to both the disease and different related treatments that influence the infection profile of patients with HMs. Future data highlighting the differences between bacterial, fungal, and viral infection distribution with higher statistical power might be useful to predict patients’ infection risk and inform clinical decision making. While coronavirus disease 2019 (COVID-19) infection data were not collected in the studies analyzed, it is recognized that patients with HMs are at risk for severe COVID-19. In addition, the information gained from the use of vaccines against COVID-19 in these patients has been extremely informative in terms of providing functional vaccine response data to refine risk stratification.

Interestingly, despite both B-lineage monoclonal antibodies against CD20 and tyrosine kinase inhibitors being detrimental to B-cell development, hypogammaglobulinemia was detected only in patients with CLL who received ublituximab and ibrutinib (BTK) combination therapy compared with patients treated with ibrutinib monotherapy who did not show a decrease in their IgG serum concentration. Notably, neutropenia, pneumonia, bronchitis, and *Herpes zoster* infections were also higher in patients treated with ublituximab and ibrutinib combination therapy compared with ibrutinib monotherapy (62). It is possible that monotherapy has been used in less severe disease settings and that a balance exists between immunosuppression from the therapy on normal immune cells and reduction in tumor-related immunosuppression due to the therapy.

This systematic literature review has several limitations: i) as not all the studies analyzed specified precise definitions for hypogammaglobulinemia, infections and SAD, this might have influenced the data as slightly different outcomes may have been captured; ii) systematic literature reviews are not powered to have statistical significance; therefore, data should be considered as exploratory. However, they can help highlight trends and possible correlations that lay the foundation for further studies; iii) most of the data came from phase III clinical trials, which do not necessarily reflect real-life clinical practice (147). Some investigators recognize the pivotal role of real-world data and evidence that can be optimized (148, 149). Meta-analysis of data from hematological databases is one avenue that could provide insightful follow-up to extrapolate information on the rate of patients with HMs and hypogammaglobulinemia due to various cancer treatments in real-life settings; iv) finally, these drugs may be used at various times throughout a disease course and as induction or maintenance therapy. Therefore, as the risk of infection can vary depending on both the timing from diagnosis and severity of disease, direct comparison of infections rates between drugs must be undertaken with caution since data were not normalized for time exposure to agents and infection reporting. Future analyses will be crucial in evaluating the rates of hypogammaglobulinemia and infections in early versus late disease course. Moreover, distinction between BTK and phosphoinositide 3-kinase (PI3K) inhibitors were not performed due to the low number of studies that tested PI3K inhibitors, therefore an overall kinase inhibitor category was used, which may limit practical application of data from this category.

With this systematic literature review, the authors wish to shed light on which treatments might contribute to the development of SID in this rapidly evolving therapeutic area and to highlight the importance of reporting data on hypogammaglobulinemia, both before and during therapy in patients with HMs. The authors believe that, while treatment of the malignancy is clearly of primary importance, there are still several knowledge gaps on the management of SID (**Table 1**); therefore, efforts need to be undertaken to improve awareness of how to diagnose and treat patients with hypogammaglobulinemia, CID, and infections in HMs, as well as optimize treatments to prevent recurrent and severe infections. Without increased recording and reporting of Ig levels in this patient population, the benefits of a range management strategies such as infection exposure mitigation strategies, vaccination, antibiotics, antiviral drugs, and immunoglobulin replacement therapy (IgRT) cannot be fully evaluated (41).

## Data availability statement

The datasets presented in this study are the results of analyses conducted with data taken from online publications. The name of the publications can be found in the reference list.

## Author contributions

All authors contributed equally to the review approach/design, consensus meetings, recommendations, interpretation of literature, writing, and critical review of this article. All authors reviewed the final manuscript and agreed on the decision to submit it for publication.
