# Supplementary figures and images for: Agents contributing to secondary immunodeficiency development in patients with multiple myeloma, chronic lymphocytic leukemia and non-Hodgkin lymphoma: A systematic literature review
Source: Front Oncol. 2023 Feb 7;13:1098326. doi: 10.3389/fonc.2023.1098326 (PMC9941665; doi:10.3389/fonc.2023.1098326)

**Supplementary Figure 1. PRISMA flow diagram**

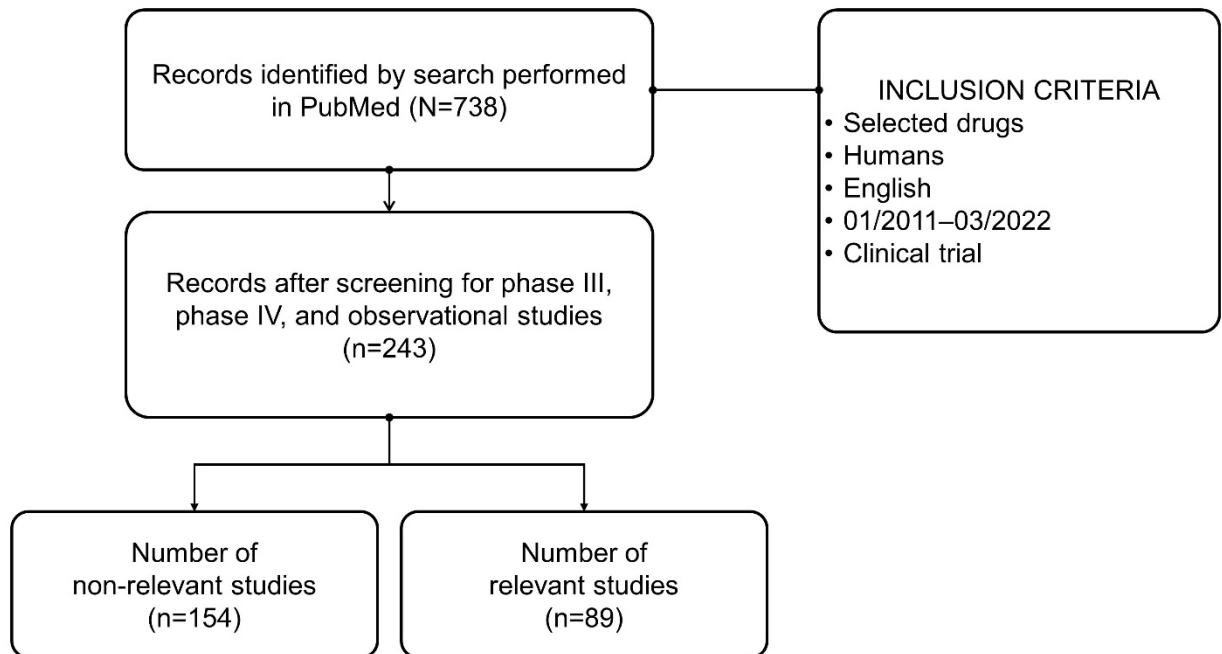

Supplement: Supplementary file 1 [file Image_1.pdf]
