# Supplementary material for: Agents contributing to secondary immunodeficiency development in patients with multiple myeloma, chronic lymphocytic leukemia and non-Hodgkin lymphoma: A systematic literature review
Source: Front Oncol. 2023 Feb 7;13:1098326. doi: 10.3389/fonc.2023.1098326 (PMC9941665; doi:10.3389/fonc.2023.1098326)
Supplement: Supplementary file 2 [file Table_1.docx]

**Supplementary Table 1. Proportions of patients with CLL, MM, and NHL who had infections (any grade and grade ≥3), neutropenia (any grade or grade ≥3), or hypogammaglobulinemia divided per year groups.**

| **Malignancies** | **Studies (n)** | **Any grade neutropenia^*^** | | **Grade ≥3 neutropenia^*^** | | **Any grade infections^*^** | | **Grade ≥3 infections^*^** | | **Hypogamma^*^** |
| --- | --- | --- | --- | --- | --- | --- | --- | --- | --- | --- |
|  |  | **Mean** | **Range (%)** | **Mean** | **Range (%)** | **Mean** | **Range (%)** | **Mean** | **Range (%)** | **Range (%)** |
| **2011–2016** | | | | | | | | | | |
| CLL | 4 | 23.4 | 17.6–27.8 | 13.2 | 3.0–27.2 | 43.9 | 41.9–45.8 | 11.4 | 9.2–13.0 | – |
| MM | 16 | 51.3 | 19.6–85.5 | 27.8 | 6.3–80.0 | 22.9 | 0.0–68.0 | 12.4 | 0.0–34.0 | – |
| NHL | 14 | 32.6 | 3.4–78.3 | 32.8 | 2.4–81.5† | 26.6 | 4.0–53.3 | 7.5 | 1.8–23.0 | 5.9 |
| Total | 34 | 38.9 | 3.4–85.5 | 27.5 | 2.4–81.5 | 27.0 | 0.0–68.0 | 10.8 | 0.0–34.0 | 5.9 |
| **2017–2022** | | | | | | | | | | |
| CLL | 13 | 39.8 | 9.4–64.0 | 34.8 | 4.1–60.0 | 53.1 | 14.4–69.1 | 21.1 | 6.4–39.0 | 0–15.3 |
| MM | 22 | 28.9 | 9.8–71.0 | 19.6 | 2.0–54.1 | 48.8 | 29.0–59.4 | 21.0 | 3.7–50.2 | – |
| NHL | 20 | 36.9 | 3.2–87.5 | 43.6 | 0.0–100.0 | 35.6 | 11.5–81.0 | 13.6 | 0.9–38.0 | – |
| Total | 55 | 34.7 | 3.2–87.5 | 30.9 | 0.0–100.0 | 44.2 | 11.5–81.0 | 18.6 | 0.9–50.2 | 0–15.3 |

*The reporting criteria for time to adverse events differed across studies.
Neutropenia grades: grade 1, less than the lower limit of normal–1,500 per mm^3^; grade 2, 1,499–1,000 per mm^3^; grade 3, 999–500 per mm^3^; grade 4, <500 per mm^3^; grade 5, death.
Infection grades: grade 1, –; grade 2, localized, local intervention indicated; grade 3, IV antibiotic, antifungal, or antiviral intervention indicated, interventional radiology or operative intervention indicated; grade 4, life-threatening consequences e.g., septic shock, hypotension, acidosis, or necrosis; grade 5, death.
CLL, chronic lymphocytic leukemia; hypogamma, hypogammaglobulinemia; IV, intravenous; MM, multiple myeloma; NHL, non-Hodgkin lymphoma; –, not reported.
